# “I Am Like a Lost Child”: L2 Writers' Linguistic Metaphors as a Window Into Their Writer Identity

**DOI:** 10.3389/fpsyg.2021.648667

**Published:** 2021-04-08

**Authors:** Shizhou Yang, Yinyin Peng

**Affiliations:** ^1^English Communication Department, International College, Payap University, Chiang Mai, Thailand; ^2^East Asian Studies, School of Language, Culture and Society, University of Leeds, Leeds, United Kingdom

**Keywords:** metaphor, identity, writing, foreign language, self perception

## Abstract

The past two decades have witnessed a burgeoning literature on L2 writers' identities, especially their discoursal identities. In contrast, little attention is paid to the writers' felt sense of self when they write in an L2, which is an integral dimension of their autobiographical self. In this article, we provide empirical evidence of the nature of this aspect of L2 writer identity. To illustrate, we analyzed linguistic metaphors elicited from three groups of L2 writers (*N* = 83), majoring respectively in Thai, Japanese, and English in a Chinese university. Descriptive analysis shows that, due to challenges in content, language, organization, and cultural differences, a majority of L2 writers, especial Thai and Japanese L2 writers, experience a diminishing sense of self when they write in L2. In contrast, some L2 writers, especially English L2 writers, find writing in an L2 liberating, revealing the impact of their individual learning trajectories and pedagogical practices on L2 writers' felt sense of self. Findings suggest that L2 writers' identity work is both complex and dynamic. L2 writing teachers can utilize the metaphor questionnaire as a tool to facilitate their learner needs analysis and to raise L2 writers' metacognition.

## “I Am Like a Lost Child”: L2 Writers' Self-Perceptions

### Introduction

Second language or L2 writers' identity has become a prominent issue among L2 writing and composition scholars in the past two decades (Harklau, 2000; Hyland, 2002, 2012; Li, 2007; Cox et al., 2010; Liu, 2011; Yang, 2013; Canagarajah, 2015, 2020; Zhao, 2015). Based on her analysis of published L2 memoires, Li (2007) claimed that all bilingual writers go through two phases of belonging in their relationships with their multiple languages and cultures. In the initial phase, bilingual writers are “souls in exile,” who are removed from both home and host languages and cultures. In the latter phase, bilingual writers may become “global souls,” who synergize their home and host languages and cultures in their language use. Therefore, L2 writing is gradually recognized as a process of L2 writers inventing their new textual identities in relation to their linguistic backgrounds and within each particular context of writing (Cox et al., 2010; You, 2011).

Yet it seems absurd to assume that all L2 writers begin as estranged beings from L1 and L2 languages and cultures, and end as celebrants of both. After all, much L2 writing occurs in contexts where the writers have not crossed national boundaries, nor have they experienced any acute change of daily life because they write in an L2. Besides, only a handful of L2 writers become published writers. As such, the majority of L2 writers who learn to write in an L2 within their home countries, who are less proficient in L2 writing, and who do not write to publish, may need additional points of reference to locate themselves in their own sociocultural world. Assuming that identity is relational (Norton, 2000) and that autobiographical self entails a writer's sense of self-worth (Ivanic, 1998), we consider that these L2 writers always write in an L2 with a personally meaningful sense of self. We further claim that such a sense of self can be revealed by the linguistic metaphors employed by L2 writers to describe themselves when they write in a particular L2 in a particular context. Our study of three groups of L2 writers provides empirical evidence of the link between L2 writers' metaphors and their self-perceptions.

### Literature Review

#### L2 Writer Identity

Ivanic's (1998) three-dimensional sociocultural framework is pivotal to existing research on L2 writer identity. The first dimension focuses on *autobiographical self*, i.e., the writing person, his or her evolving life history and accompanying as well as still emergent sense of self (p. 24), which shape both L2 writers' writing processes and texts (e.g., Hirvela and Belcher, 2001; Zhao, 2015). The second dimension is *discoursal self*, i.e., impressions of the writer, also known as “textual identities” (Lam, 2000), as projected through a text's discourse features (Ivanic, 1998, p. 25), such as the use of pronouns, lexicons, and citations (Helms-Park and Stapleton, 2003; Canagarajah, 2015; Zhao, 2015). The third dimension features *self as author*, i.e., the textual positioning of the writer as the originator of content (Ivanic, 1998, p. 26), which foregrounds L2 writers' pronoun use, stance-taking, and text-borrowing practices in academic writing (Hyland, 1997, 2012; Ouellette, 2008).

As Ivanic (1998) cautions, all these three dimensions of writer identity are interconnected and shaped by the *possibilities of self-hood* in each specific context of writing (pp. 27–29). This attention to writing contexts helps to L2 writing scholars to address issues such as imbalanced power relations between native and non-native speakers of English, sociopolitical and disciplinary preferences for certain genres and textual practices (Hyland, 2002; You, 2010), institutional ways of positioning L2 writers (Harklau, 2000), and language ideologies in academic contexts (Canagarajah, 2015, 2020).

From a constructivist perspective at least, L2 writers' identity work may occur at two broad levels: social and personal. Socially, L2 writers may be positioned by institutions, as commonly done in the ESL context, in terms of one of these labels: “non-native speaker of English,” “ESL,” “EFL,” “multilingual,” “emergent bilingual,” “biliterate,” and “translingual.” Personally, each L2 writer may relate to any of these labels in light of his or her own experiences, preferences, and aspirations (cf., Shuck, 2010).

This kind of identity work with given labels can be well-illustrated by a recent publication, *Autoethnography in ELT* edited by Yazan et al. (2021). Most of the English language teachers, who write in English as an L2, reflected on their experiences of learning and teaching English, and, in one way or another, rejected the deficit-oriented identity of a “non-native speaker” and embraced a resource-oriented transnational and translingual identity. Thus, L2 writers, who by the nature of their engagement of writing in an L2, can textually explore, externalize, and display their “trope of becoming” translingual writers (Canagarajah, 2020) or “global souls” (Li, 2007) through writing about their own literacy experiences.

Although studies like those reviewed above suggest both the challenges and opportunities associated with writing in an L2, we are far from knowing fully “the hidden conceptual and emotional world of the individual” (Hanauer, 2004, p. 4) L2 writers, particularly those who learn to write in an L2 as a foreign language. Seen from Ivanic's (1998) sociocultural framework, the umbrella term “L2 writer” provides only a starting point for unpacking the complex and dynamic nature of L2 writers' identity work. Furthermore, existing studies on L2 writer identity have mainly attended to discursive self and self as author, particularly associated with academic writing in English as a second language (ESL). In contrast, scarce literature is available on L2 writers' autobiographical self that features their felt sense of self or self-perception when they write in an L2 within a foreign language context. We are yet to learn more about an L2 writer as “a subject in process” (Kramsch, 2003, p. 3) within their own contexts of writing.

#### Metaphor and Identity

Traditionally, metaphor refers to a linguistic expression of similarity between two concepts. According to Lakoff and Johnson (1980), it is basically about “understanding and experiencing one kind of thing in terms of another” (p. 5). With numerous examples from daily lives, Lakoff and Johnson (1980) argued that metaphors go beyond the language itself to shape human thought and ways of forming concepts. Lakoff (1993) further concluded that metaphors are pervasive, capable of revealing abstract ideas, and conceptual in nature (pp. 40–41). Lakoff and Johnson's (1980) position on metaphor, also known as the Conceptual Metaphor Theory, assumes that any metaphorical use of language always points to some unstated concept behind (Kövecses, 2017). Furthermore, metaphor use, as in the case of *Life is a journey*, maps the concrete source domain (journey) to a more abstract target domain (life) (Kövecses, 2020, p. 5).

However, later research shows that linguistic metaphors cannot be equated to conceptual metaphors. Or vice versa. A linguistic metaphor refers to any metaphoric use of language that suggests any resemblance between two entities. It does not promise any systematic mapping between the two conceptual domains, as typical of conceptual metaphors. Through his similarity and proximity experiments, Casasanto (2009), for instance, finds that linguistic metaphors show but partial knowledge of people's mental representations of abstracts concepts such as time, space, and speed. In other words, people's sense of similarity may be shaped by factors such as speed, time, and space. Similarly, complete and systematic matching between two conceptual domains is close to impossible. For instance, the building metaphor for theory seems to equate a theory (target domain) to a house (source domain) but a house may have a cell, whose equivalent is not found in a theory.

Despite critiques, metaphor analysis has been found a productive way to understand language learners' identity work. As earlier metaphor-based studies (e.g., Kramsch, 2003; Huang, 2011) illustrate, language learners' metaphors provide *emic*, vivid and rich descriptions of their understandings of themselves in relation to language learning. Who they are when learning a new language (the target domain) can be understood, at least in part, through their linguistic metaphors (the source domain) that describe their language learning experiences. Therefore, metaphors not only provide a window into learners' learning (Crick and Grushka, 2009), but also their language-mediated identity construction processes (e.g., Kramsch, 2003). As Alsup (2006) suggests, metaphor is “a powerful form for identity creation and a catalyst for personal growth” (p. 10).

The same can be argued about metaphor analysis and L2 writers' identity work (Huang, 2011; Wan, 2014). Increasing literature on the subject shows that L2 writing is not just a process of acquiring some mechanic writing skills. Rather, it is a process of self-invention and re-configuration in a given community of practice (Cox et al., 2010; Yang, 2013, 2020). Instead of relying on labels such as ESL, which may not be relevant in a foreign language environment after all, we turn to L2 writers' own metaphors associated with writing in an L2. Accordingly, we asked the following three questions:

1. To what extent do L2 writers' metaphors suggest a negative relationship with L2 writing?
2. To what extent L2 writers do L2 writers' metaphors suggest a positive relationship with L2 writing?
3. What factors contribute to the L2 writers' use of particular types of metaphors to represent themselves?

## Methods

Data were collected through a one-sentence metaphor-eliciting questionnaire: “When I write in … (a particular L2, i.e., Thai, Japanese, or English), I am like … because….” The questionnaire was distributed to three groups of university students, majoring respectively in Thai, Japanese, and English at a culturally and linguistically diverse university in China. For both the Thai and Japanese majors, who did not begin learning their respective L2s until college, the questionnaire was written in Chinese with instructions that the findings of study may be helpful to improve college students' learning effectiveness. The questionnaire also asked students' background information such as gender, age, and ethnicity. Some students identified themselves as ethnic minority backgrounds, such as Lahu, implying that their home language might be some ethnic language. Nonetheless, as customary in contemporary Chinese education, Mandarin is usually the language of instruction and it can be assumed that all these students, who had successfully passed the competitive College Entrance Examination, possessed a high level of proficiency in Chinese. Both Thai and Japanese L2 writers responded in Chinese anonymously. These responses were translated into English and checked by the two authors, who are both proficient in Chinese and English. Thai L2 writers' data are referred to as T1, T2, T3, etc. and Japanese L2 writers' data are referred to as J1, J2, J3, etc.

For the English L2 writers, who had studied English in China for at least 7 years, the questionnaire was written in English. All the responses were in English and the participants also wrote their names. However, during the analysis, all data were anonymized, with English L2 writers' data referred to as E1, E2, E3, etc.

Convenience sampling was used. That is, Author B asked her friend to distribute the questionnaire among the Thai and Japanese major students. Author A distributed the questionnaire among the English major students he was teaching. All participants were from their own intact classes. A total of 83 questionnaires were distributed and collected. Among them, 34 majored in English, 21 majored in Japanese, and 28 majored in Thai. When participating in this study, the Japanese majors (juniors) had just begun taking a writing course. The Thai majors were seniors who had completed their academic writing course in Thailand in their junior year. All the participants were around 20 years old.

Data analysis included three stages. First, we identified nine invalid questionnaires from the Thai L2 writers, who did not actually create any linguistic metaphor. An example is: “When I write in English, I like *to express my own feelings* instead of imagining some nonexistent things…” These questionnaires were excluded from further analysis. Thus, the total valid questionnaires from the Thai L2 writers were 19.

Next, we categorized metaphors based on the kind of relationship they indicate that L2 writers have with L2 writing, i.e., positive or negative or neutral. See examples below:

Example of a positive metaphor:

When I write in English, I feel like I am a superman… (E30)

Example of a negative metaphor:

When I write in English, I feel like a dumb person… (E9)

Example of a neutral metaphor:

When I write in English, I feel like a searcher… (E8)

The distinction between these negative and positive metaphors has precedents in earlier studies. Evoking height as the main concept, for instance, people may use “high” as a positive metaphor to suggest “good” whereas “low” as a negative metaphor to suggest “bad” (Goatly, 2011, p. 31).

Caution was exercised not to rely on linguistic metaphor alone to reveal the nature of relationship. Thus, in the second stage, we also analyzed the “co-text” (Reijnierse et al., 2020), i.e., the texts that surround a given linguistic metaphor, to confirm if it should be treated as suggesting a negative or positive relationship. This is important because L2 writers may use the same metaphor for different functions. Take the linguistic metaphor of “a fish” as an example.

When I write in English now, I am like a fish because I find a river that belongs me. In this river I swim freely and I realize my item is the ocean. (E26)

*Speaking of writing in Japanese, I feel like I am a fish, swimming around, unable to grasp what is important*… (J5, italicized texts are our translations from Chinese)

As seen through the co-texts, whereas E26 evokes a “fish” metaphor to suggest a sense of freedom and belonging, J5 uses the same metaphor to suggest a sense of inability or lack of control. Thus, E26 was categorized as a “positive” metaphor whereas J5 was categorized as “negative” metaphor. Paying attention to co-texts assisted us to determine the nature of relationship more accurately.

Both authors identified the metaphors of English L2 writers independently, with an initial interrater reliability of 0.79. We then cross-checked each other's categorization, resolved any disagreement through discussion, reached an interrater reliability of 1.0. We then applied the same procedure to analyzing the other two groups of L2 writers' metaphors.

Last, we analyzed reasons why L2 writers used particular metaphors to represent themselves. Here, our focus was on the co-texts of L2 writers' metaphors, i.e., the explanation followed by *because*.

## Results and Discussions

### Types of Metaphors

#### Negative Metaphors

The general trend is that L2 writers' metaphors suggest that the writers have developed a negative relationship with L2 writing. That is, these L2 writers express a depreciating sense of self, as represented by their metaphors. For the Thai L2 writers, for instance, 15 out of 19 or 78.9% produced negative metaphors such as:

*When I write in Thai, I'm like a one-legged bird. I'm also like a junior high school student…* (T3)

*When I write in Thai, I feel like a piece of drifting leaf…*(T4)

*When I write in Thai, I am like a junior school student…*(T27)

Other self-depreciating metaphors used by Thai L2 writers include: “a primary school student,” “a foreigner,” “a kid in the kindergarten,” “an old man,” “a babbling baby,” etc. In fact, 14 of the Thai L2 writers evoked a human image, except that in all these cases, they emphasize that their age and intellect became lower.

Compared to the Thai L2 writers, an even larger percentage of Japanese L2 writers, 18 out 20 or 90% of them, experience a depreciating sense of self when writing in Japanese L2. They used a wider range of metaphors, including both other humans and inanimate objects, than the Thai L2 writers:

*When I write in Japanese, I feel like an anti-Japanese soldier…* (J1)*When I write in Japanese, I feel like a file compressing software…* (J8)

*When I write in Japanese, I feel like Doraemon (a Japanese cartoon character who is afraid of mice) dreaming of running into a mouse…* (J12)

Other self-depreciating metaphors used by Japanese L2 writers include: “*a pupil*,” “*a drunken Wu Song*,” “*an inexperienced driver*,” “*a language-impaired person*,” etc. Some of them such as “*a low-class animal*” (J4) and “*a dog*” (J20) carry a very negative connotation particularly within a Chinese cultural context.

As for the English L2 writers, self-depreciating metaphors also abound although the percentage is much lower. Of the 34 questionnaires, a quarter (26%) employed negative metaphors such as the following:

When I write in English now, I am like a lost child… (E27)

When I write in English now, I am like a chicken with its head cut off… (E7)

When I write in English, I'm like close circuit robot… (E34)

English L2 writers also used other metaphors such as “a fish,” “a bird,” “a monkey” to foreground their changed state when they write in an L2.

#### Positive Metaphors

In rare cases, the Thai and Japanese L2 writers' metaphors suggest that the writers have developed a positive or self-empowering relationship with L2 writing. Among them, Thai L2 writers produced three positive metaphors of themselves as “*a bird*,” “*a key*,” and “*a floating cloud*.” The percentage is 13.6%. Similarly, the Japanese L2 writers only produced two positive metaphors (“*a bird*” and “*a potential winner of a Nobel Prize*”). The percentage is 10%.

In contrast, the English L2 writers have the highest percentage of positive metaphors, which were provided by 18 or 55% of the 33 participants. See, for example:

When I write in English now, I am like a superman… (E30)

When I write in English now, I am like a thoughtful person… (E3)

When I write in English now, I am like a dancer… (E15)

When I write in English now, I am like a river… (E1)

Both human images and other images are evoked. Five English L2 writers used “a bird” metaphor. Other positive metaphors include “a fish,” “a writer,” “a proser,” and the like.

#### Neutral or Conflicting Metaphors

Of all three group of participants, only the English L2 writers produced neutral or conflicting metaphors. These metaphors include:

When I write in English now, I feel like a telegraph… (E21)

When I write in English now, I feel like a searcher… (E8)

When I write in English now, I feel like a poet…I also feel like a person who gets lost in the desert… (E2)

When I write in English now, I feel like a kid trying out a bike… (E10)

When I write in English now, I feel like a traveler… (E19)

When I write in English now, I feel like a by stander… (E22)

These trends in metaphor use among the three groups of L2 writers will be discussed next in relation to the factors that contribute to their use.

### Contributing Factors

Analysis of the metaphors' co-texts further reveals factors that contribute to the L2 writers' use of particular metaphors. For the negative metaphors, the factors can be broadly grouped as language, content, and culture.

#### Causes for Negative Metaphors

Writing in an L2 entails a great demand on the writer's knowledge of lexicons and grammar. With limited proficiency, L2 writers may struggle in how to express their ideas in an L2. This is evident in the following responses from the Thai and Japanese L2 writers:

*When I write in Thai, I feel like a tiny little bird, which wants to fly high but cannot, because I have a small vocabulary, and my grammar is not good*. (T6)

*When I write in Thai, I feel like a middle school student, because all I write is simple composition, which hardly has long compound sentences with modifiers*. (T17)

*When I write in Japanese, I feel like a dog that looks at the Kana submissively and helplessly*. (J18)

*When I write in Japanese, I feel like a primary school student, who cannot say anything in a complete sentence and whose writing is filled with simple sentences*. (J14)

These L2 writers' explanations suggest that, consciously or not, L2 writers may develop their sense of self as L2 writers in relation to their existing abilities in their L1s. Their relative lack of expressive resources in an L2 may initiate in them negative self-conceptions, which feature a lowered educational level, age, and intellectual development.

The other contributing factor is challenges in generating content. L2 writing is a process of writing about something and of L2 writers figuring out in each new context what can be written, what is worth writing, and what they can write. This challenge seems especially acute with the following L2 writers, who wrote:

*When I write in Thai, I feel like a floating leaf, not knowing where to begin*. (T3)

*When I write in Japanese, I feel like Wang Ziyi, who has never gone to school. I can't think of anything. I don't know anything. My mind is completely blank*. (J13)

When I write in English now, I am like a baby because I think I don't know nothing about write [*sic*] in English, I need more idea like the sea surge out. (E7)

Additionally, cultural differences in ways of thinking and expression also contributed to some L2 writers' use of negative metaphors. Consider the following two examples:

*I don't feel anything special [when I write in Thai]. It's just applying what I have learned. However, if I compare my writing with Thai people's writing, I feel that I'm like a middle school student or a primary school student*. (T10)

*When I write in Japanese, I feel like a person with language impairment. Because there are many differences between Japanese and Chinese ways of expression, it is inevitable to use Chinese way of thinking and some expressions just can't express my ideas clearly*. (J17)

These examples showcase that L2 writers may reference their L1 counterparts in their self-conceptions.

The dominance of negative metaphors among the Thai and Japanese L2 writers may have also been influenced by the prompt they had received. The prompt implied that responses given might be used to help both current participants and future students. It may have triggered the Thai/Japanese L2 writers to mainly discuss their challenges/issues with writing. In contrast, the questionnaires for the English L2 writers were distributed in an ongoing writing class, without any explicit explanation about the future use of the study results. This difference in prompts needs to be kept in mind when interpreting the Thai/Japanese L2 writers' metaphors.

#### Causes for Positive Metaphors

With only a few cases, Thai and Japanese L2 writers' positive cases were mainly associated with a freewriting state.

*When I write in Thai, I feel like a bird, feeling very free, because I can say whatever I want, and I'm not afraid of making mistakes*. [T25]

*When I write in Thai, I feel like a key, which is unlocking the gate to knowledge. Writing is the same as speaking. Our mother-tongue is not Thai, so it's inevitable to make mistakes. Only by doing a lot of practice can we improve our level and can we correct mistakes pointed out by others*. [T26]

*When I write in Thai, I feel like a drifting cloud going wild. I write whatever comes to my mind*. (T27)

*When I write in Japanese, I feel like a bird that's free. I can say whatever I want*. (J20)

A similar state of freedom was also expressed in several English L2 writer's metaphors and their co-texts. However, unlike the Thai and Japanese L2 writers, they associated with the pedagogical context and compared with their former selves more explicitly. Consider the following examples:

When I write in English now, I am like a superman. For the first time I writed [*sic*] in English, I felt like I was a baby who couldn't say a word, walk and crawl. I failed to organize a complete composition. I didn't know how to write an advanced sentence and utilize advanced words. Grammar was not my strength, there used to be countless mistakes in my composition. But now, I am like a superman. Writing is easy to me what eat [*sic*] is to human beings. I master some complex structures of sentences. Using advanced words isn't a big deal. (E30)

When I write in English now, I am like a dancer who can ignore others when he or she is dancing. The reason why I have a such feeling is I really enjoy writing in English now. I have a thought I can write well than before. First, I know how to write the first paragraph, even I can use some interesting or meaningful sentence to attract readers' eyes. Second, I understand how I should organize the whole article. Third, I can express what I want to say at the ending of the article. (E15)

When I write in English now, I am like a middle school students. Because I am not fear as my first write when I first attended this class. I am like a children because I don't know how to write or even don't know how to use easy sentence. Before I attended this class, I written by using some 固定句型 [fixed sentence patterns] taught by our high school teacher. However, though I can't [write] some good [essays], I can express feelings from the bottom of my heart. (E11)

Note that the English L2 writers filled in their questionnaires in class after they have taken a writing course for 7 weeks. Their growing proficiency thus loomed large as they conceptualized themselves in relation to L2 writing.

## Conclusion and Implications

To conclude, the three groups of L2 writers' metaphors revealed their self perceptions when they write in their respective L2s. More specifically, regardless of their language backgrounds and years of learning English as an L2 (and in some cases, as an L3), a majority of the L2 writers manifest a depreciating sense of self with less humanity, biological age, and/or intellectual power when writing in an L2. Even when they describe themselves in human image-based metaphors, what predominates over their metaphors remains a sense of less-than self due to the various challenges in the L2 writing, concerning language use, cultural difference, and content generation. Further, a comparison, whether explicit or implicit, with their relative ease in L1 writing also accentuates their sense of less-than identity. We believe that this finding contributes to growing knowledge about the psychological burden of shame and other long ignored affects associated with language learning (Kövecses, 2000; Benesch, 2012; Liyanage and Canagarajah, 2019).

Another main finding of this study is that positive metaphors were also used. This was especially true with the English L2 writers, who have had the longest time of learning an L2. Importantly, these L2 writers mainly situated their metaphors in relation to the pedagogical context and their former autobiographical self. Thus, growing proficiency often became a trigger for positive metaphors such as “I am like a superman.” L2 writers' linguistic metaphors thus provided a situated sense of self rather than an abstract, i.e., uncontextualized, notion of self. Taken both the negative and positive metaphors together, it seems fair to conclude that L2 writers' linguistic metaphors offer a window into their writer as they change and mature.

Although these findings may not be directly translated to useful pedagogical practice, they have two major implications. First, metaphor elicitation can be an effective way to facilitate L2 writers' self-reflection. Second, L2 writing teachers may utilize the questionnaire survey to know their students better, especially in terms of where they are on their journeys as L2 writers and what challenges they are facing.

Limitations of this study are obvious. First, the categories of L2 writers are still not diverse and representative enough. Future studies may consider expanding to other types of L2 writers, who write in an L2 other than Thai, Japanese, and English, or who write in these L2s at a younger or older age. Future studies may also employ random sampling to test the findings from this study. Furthermore, this study only focuses on the L2 writers' metaphors at a particular moment and there might be some crosslinguistic issue to compare metaphors elicited in L1 Chinese and L2 English. Future studies may consider using the questionnaire several times with the same group of L2 writers, using both their L1 and L2, and thus reveal the extent to which L2 writers' linguistic metaphors become conceptual metaphors that they live by. Additionally, this study did not include other data such as classroom observation, interview, and L2 writing samples. Future research may consider expanding the data sets to explore further L2 writers' linguistic metaphors and situated identity work.

## Data Availability Statement

The raw data supporting the conclusions of this article will be made available by the authors, without undue reservation.

## Ethics Statement

Ethical review and approval was not required for the study on human participants in accordance with the local legislation and institutional requirements. Written informed consent for participation was not required for this study in accordance with the national legislation and the institutional requirements.

## Author Contributions

SY contributed to the research idea and design, data collection, analysis, writing, and revision. YP contributed to the data collection, analysis, and writing. All authors contributed to the article and approved the submitted version.

## Conflict of Interest

The authors declare that the research was conducted in the absence of any commercial or financial relationships that could be construed as a potential conflict of interest.

## References

[B1] Alsup J. (2006). Teacher Identity Discourses: Negotiating Personal and Professional Spaces. Mahwah, NJ: L. Erlbaum Associates.

[B2] Benesch S. (2012). Considering Emotions in Critical English Language Teaching: Theories and Praxis. New York, NY: Routledge.

[B3] Canagarajah A. S. (2015). “Blessed in my own way”: pedagogical affordances for dialogical voice construction in multilingual student writing. J. Second Lang. Writ. 27, 122–139. 10.1016/j.jslw.2014.09.001

[B4] Canagarajah A. S. (2020). Transnational Literacy Autobiographies as Translingual Writing. New York, NY: Routledge.

[B5] Casasanto D. (2009). When is a linguistic metaphor conceptual metaphor? in Human Cognitive Processing, Vol. 24, eds Evans V. Pourcel S. (Amsterdam: John Benjamins Publishing), 127–145. 10.1075/hcp.24.11cas

[B6] Cox M. Jordan J. Ortmeier-Hooper C. Swartz G. G. (eds.). (2010). Reinventing Identities in Second Language Writing. Urbana, IL: NCTE Press.

[B7] Crick R. D. Grushka K. (2009). Signs, symbols and metaphor: linking self with text in inquiry based learning. Curric. J. 20, 447–464. 10.1080/09585170903425069

[B8] Goatly A. (2011). Metaphor as resource for the conceptualisation and expression of emotion, in Affective Computing and Sentiment Analysis: Metaphor, Ontology, Affect and Terminology, ed Ahmad K. (London: Springer), 19–32.

[B9] Hanauer D. I. (2004). Poetry and the Meaning of Life. Toronto, ON: Pippin.

[B10] Harklau L. (2000). From the “good kids” to the “worst”: representations of english language learners across educational settings. TESOL Q. 34, 35–67. 10.2307/3588096

[B11] Helms-Park R. Stapleton P. (2003). Questioning the importance of individualized voice in undergraduate L2 argumentative writing: an empirical study with pedagogical implications. J. Sec. Lang. Writ. 12, 245–265. 10.1016/j.jslw.2003.08.001

[B12] Hirvela A. Belcher D. (2001). Coming back to voice: the multiple voices and identities of mature multilingual writers. J. Second Lang. Writ. 10, 83–106. 10.1016/S1060-3743(00)00038-2

[B13] Huang W. C. (2011). The EFL learner identity development: a perspective of metaphor. Int. J. Innov. Interdiscip. Res. 1, 1–13.

[B14] Hyland K. (1997). Qualification and certainty in L1 and L2 students' writing. J. Second Lang. Writ. 6, 183–205. 10.1016/S1060-3743(97)90033-3

[B15] Hyland K. (2002). Options of identity in academic writing. ELT J. 56, 351–358. 10.1093/elt/56.4.351

[B16] Hyland K. (2012). Undergraduate understandings: stance and voice in final year reports, in Stance and Voice in Written Academic Genres, eds Hyland K. Guinda C. S. (New York, NY: Palgrave Macmillan), 134–150.

[B17] Ivanic R. (1998). Writing and Identity: The Discoursal Construction of Identity in Academic Writing. Amsterdam: John Benjamins.

[B18] Kövecses Z. (2000). Metaphor and Emotion Language, Culture, and Body in Human Feeling. Cambridge: Cambridge University Press.

[B19] Kövecses Z. (2017). Conceptual metaphor theory, in The Routledge Handbook of Metaphor and Language, eds Semino E. Demjén Z. (New York, NY: Routledge), 13–27.

[B20] Kövecses Z. (2020). Extended Conceptual Metaphor Theory. Cambridge: Cambridge University Press.

[B21] Kramsch C. (2003). Metaphor and the subjective construction of beliefs, in Beliefs About SLA, Vol. 2, eds Kalaja P. Barcelos A. M. F. (New York, NY: Springer), 109–128. 10.1007/978-1-4020-4751-0_5

[B22] Lakoff G. (1993). The Contemporary Theory of Metaphor. Berkeley, CA: UC Berkeley. Available online at: https://escholarship.org/uc/item/54g7j6zh (accessed February 17, 2021).

[B23] Lakoff G. Johnson M. (1980). Metaphors We Live By. Chicago, IL: University of Chicago Press.

[B24] Lam W. S. E. (2000). L2 literacy and the design of the self: a case study of a teenager writing on the internet. TESOL Q. 34, 457–482. 10.2307/3587739

[B25] Li X. M. (2007). Souls in exile: identities of bilingual writers. J. Lang. Identity Educ. 6, 259–275. 10.1080/15348450701542256

[B26] Liu Y. (2011). Power perceptions and negotiations in a cross-national email writing activity. J. Sec. Lang. Writ. 20, 257–270. 10.1016/j.jslw.2011.06.001

[B27] Liyanage I. Canagarajah S. (2019). Shame in English language teaching: desirable pedagogical possibilities for Kiribati in neoliberal times. TESOL Q. 53, 430–455. 10.1002/tesq.494

[B28] Norton B. (2000). Identity and Language Learning: Gender, Ethnicity, and Educational Change. London: Pearson Education.

[B29] Ouellette M. A. (2008). Weaving strands of writer identity: self as author and the NNES “plagiarist. J. Second Lang. Writ. 17, 255–273. 10.1016/j.jslw.2008.05.002

[B30] Reijnierse W. G. Burgers C. Krennmayr T. Steen G. T. (2020). The role of co text in the analysis of potentially deliberate metaphor, in Drawing Attention to Metaphor: Case Studies Across Time Periods, Cultures and Modalities, eds Biase C. D. Dyson Egg M. (Amsterdam: John Benjamins), 15–38.

[B31] Shuck G. (2010). Language identity, agency, and context: the shifting meanings of multilingual in Reinventing Identities in Second Language Writing, eds Cox M. Jordan J. Cortmeier-Hooper C. Schwarts G. G. (IL: NCTE), 117–138.

[B32] Wan W. (2014). Constructing and developing ESL students' beliefs about writing through metaphor. J. Second Lang. Writ. 23, 53–56. 10.1016/j.jslw.2014.01.002

[B33] Yang S. (2013). Autobiographical Writing and Identity in EFL Education. New York, NY: Routledge.

[B34] Yang S. (2020). Meaningful literacy and agentive writer identity. MEXTESOL J. 44, 1–15.

[B35] Yazan B. Canagarajah A. S. Jain R. (2021). Autoethnographies in ELT: Transnational Identities, Pedagogies, and Practices. New York, NY: Routledge.

[B36] You X. (2010). Writing in the Devil's Tongue: A History of English Composition in China. Illinois, IL: Southern Illinois University Press.

[B37] You X. (2011). Chinese white-collar workers and multilingual creativity in the diaspora. World Eng. 30, 409–427. 10.1111/j.1467971X.2011.01698.x

[B38] Zhao Y. (2015). Second Language Creative Writers: Identities and Writing Processes. Bristol: Multilingual Matters.

